# A controlled study on the cognitive effect of alpha neurofeedback training in patients with major depressive disorder

**DOI:** 10.3389/fnbeh.2014.00296

**Published:** 2014-09-02

**Authors:** Carlos Escolano, Mayte Navarro-Gil, Javier Garcia-Campayo, Marco Congedo, Dirk De Ridder, Javier Minguez

**Affiliations:** ^1^Department of Robotics, Perception and Real Time Group, Aragon Institute of Engineering Research (I3A), University of ZaragozaZaragoza, Spain; ^2^Department of Computer Science and Systems Engineering (DIIS), University of ZaragozaZaragoza, Spain; ^3^BitBrain Technologies SLZaragoza, Spain; ^4^Department of Mental Health in Primary Care, Aragon Health Sciences Institute (IACS), University of ZaragozaZaragoza, Spain; ^5^Psychiatric Service in the Miguel Servet University Hospital, University of ZaragozaZaragoza, Spain; ^6^GIPSA-Lab, Département Images et Signal, CNRS, University of GrenobleGrenoble, France; ^7^Department of Surgical Sciences, Dunedin School of Medicine, University of OtagoNorth Dunedin, New Zealand

**Keywords:** major depressive disorder (MDD), neurofeedback (NF), EEG, cognitive enhancement, working memory (WM), upper alpha (UA)

## Abstract

Cognitive deficits are core symptoms of depression. This study aims to investigate whether neurofeedback (NF) training can improve working memory (WM) performance in patients with major depressive disorder (MDD). The NF group (*n* = 40) underwent eight NF sessions and was compared to a non-interventional control group (*n* = 20). The NF protocol aimed to increase the individual upper alpha power in the parieto-occipital area of the scalp. Main cognitive variable was WM, which was measured pre- and post- training along with other variables such as attention and executive functions. EEG was recorded in both eyes closed resting state and eyes open task-related activity, pre- and post- NF training, and pre- and post- the NF trials within each session. A power EEG analysis and an alpha asymmetry analysis were conducted at the sensor level. Frequency domain standardized low resolution tomography (sLORETA) was used to assess the effect at brain source level. Correlation analysis between the clinical/cognitive and EEG measurements was conducted at both the sensor and brain source level. The NF group showed increased performance as well as improved processing speed in a WM test after the training. The NF group showed pre-post enhancement in the upper alpha power after the training, better visible in task-related activity as compared to resting state. A current density increase appeared in the alpha band (8–12 Hz) for the NF group, localized in the subgenual anterior cingulate cortex (sgACC, BA 25). A positive correlation was found for the NF group between the improvement in processing speed and the increase of beta power at both the sensor and brain source level. These results show the effectiveness of this NF protocol in improving WM performance in patients with MDD.

## 1. Introduction

Major depressive disorder (MDD) is a severe, chronic, mood disorder characterized by episodes of sadness, loss of interest and motivation, pessimism, and suicidal thoughts (DeRubeis et al., 2008). Depression affects each year 13–14 million citizens in the United States, with a lifetime prevalence of 16.2% (Kessler et al., 2003). MDD is estimated to become the second cause of burden of disease in 2030 (Mathers and Loncar, 2006). The current standard treatment for depression is antidepressant medication. Unfortunately with this treatment about 33% of patients fail to achieve remission (Anderson et al., 2008). Moreover, the patient compliance to the treatment can decrease due to the side effects such as sexual dysfunction, gastrointestinal problems, and weight gain (Brunoni et al., 2009). For this reason, the development of new treatments is constantly explored. In particular, recently there has been a renewed interest on neuromodulation techniques such as repetitive transcranial magnetic stimulation, transcranial direct current stimulation, and neurofeedback (DeRubeis et al., 2008; Brunoni et al., 2009).

The aim of neurofeedback (NF) is to allow the subjects to self-regulate brain activity. NF consists in measuring the brain activity and providing the subjects with real-time feedback covarying with the brain patterns of interest. Thus, the subjects may acquire a certain degree of awareness of the underlying brain processes and learn to regulate them (Congedo et al., 2004). Most of the NF protocols applied to date to depressive patients are based on EEG findings of frontal asymmetry in the alpha frequency band, with depressive patients showing left hypoactivation (Henriques and Davidson, 1991; Davidson, 1998, 2004; Coan and Allen, 2004). These findings are interpreted as a dysfunction of the prefrontal cortex (PFC) and a predisposition toward negative emotions and behavioral withdrawal (Davidson, 2004; DeRubeis et al., 2008). While some studies support this theory (Gotlib, 1998; Lubar et al., 2003), controversial results have also been reported (Reid et al., 1998). Furthermore, some studies found asymmetry changes over recording sessions not related to the clinical symptoms of the patients (Allen et al., 2004b), thus its consideration as a marker for depression remains unclear to date (Allen and Cohen, 2010). Some NF studies have tried to reduce the alpha asymmetry in an attempt to alleviate the depressive symptoms, reporting promising results (Baehr et al., 1997, 2001; Hammond, 2000, 2005). However, to the best of our knowledge, none of the early studies were appropriately controlled. The first controlled study of this kind reported a reduction in depressive symptoms and an improvement in executive functions (Choi et al., 2011).

The present work is based on a different rationale. Our objective is to alleviate the cognitive symptoms of depression. Cognitive deficits are core symptoms of depressive disorders with a clear-cut impact on social and occupational functioning, with patients showing decreased performance in working memory (WM) and attention, among others (Austin et al., 2001; Castaneda et al., 2008; Gotlib and Joormann, 2010). Furthermore, depressive patients show biases in the processing of emotional contents in WM (Gotlib and Joormann, 2010; Levens and Gotlib, 2010). For example, Levens and Gotlib (2010) performed an emotion n-back WM task in which depressive and control individuals had to match the valence of stimuli (happy, neutral, or sad). Comparing the depressive vs. control individuals they found in the 2-back task that depressive individuals were slower to match emotional stimuli in WM (regardless of the valence), and they integrated faster and removed slower the sad stimuli from WM. Recent evidences thus suggest that cognitive deficits may not only be correlates of depression, but they may also increase the risk for depression (Gotlib and Joormann, 2010; Levens and Gotlib, 2010).

We hereby explore WM entrainment by means of a NF protocol aimed to up-regulate the alpha power in posterior locations of the scalp. A large body of research has highlighted the relation between alpha oscillations and WM performance through inhibitory mechanisms (Klimesch et al., 2007; Freunberger et al., 2011). Recent evidences suggest that the inhibition of irrelevant information (filtering efficiency to WM contents) is a key factor in WM performance (Vogel et al., 2005; McNab and Klingberg, 2007), whereas the neuronal substrates of inhibitory mechanisms is hypothetized to be related to alpha oscillations (Klimesch et al., 2007; Sauseng et al., 2009; Freunberger et al., 2011). For example, Sauseng et al. (2009) performed a visuospatial WM task in which a memory array displayed either on the right or left visual hemifield had to be retained. During the retention interval of the task, repetitive transcranial magnetic stimulation (rTMS) was delivered (at 10 Hz to increase alpha amplitude) in either contralateral or ipsilateral parietal sites to the items to be retained. Increased performance was found when rTMS was applied ipsilaterally to the items to be retained, suggesting that enhanced alpha oscillations effectively suppressed irrelevant information. In this line, some NF studies have reported higher cognitive performance in healthy users after regulating the alpha oscillations, specifically measured in WM (Escolano et al., 2011; Nan et al., 2012), visuospatial rotation (Hanslmayr et al., 2005; Zoefel et al., 2011), and procedural learning (Ros et al., 2014). The reader is directed to Gruzelier (2013) for a review on NF studies on cognitive enhancement.

To the best of our knowledge, this is the first NF study exploring the cognitive effect of WM entrainment in patients with MDD. Some preliminary results of the current study have been published in Escolano et al. (2013). The trained parameter was the power in the individual upper alpha band, since the upper part of alpha has been suggested to respond to cognitive demands (Klimesch, 1999) and is the direction followed by several alpha-based NF studies (Hanslmayr et al., 2005; Escolano et al., 2011; Zoefel et al., 2011; Nan et al., 2012).

## 2. Materials and methods

### 2.1. Participants

Seventy-four participants diagnosed with major depressive disorder (MDD) were allocated to the NF group (*n* = 50) or to the control group (*n* = 24). Participants were not randomly allocated to groups (see Section 4). Patients were recruited from different health centers in the city of Zaragoza (Spain). The inclusion criteria were age range (18–65 years), Spanish as the native language, diagnosis of MDD according to DSM-IV, and stable pharmacological/psychological treatment. The exclusion criteria were diagnosis of comorbid disorders (e.g., schizophrenia, drug addiction, dementia). The experimental design was approved by the Ethical Review Board of the regional health authority and followed the Declaration of Helsinki. All participants signed an informed consent. Five participants dropped out the study due to the inability to perform the cognitive assessments or recording sessions, four of whom belonging to the NF group. Among the remaining participants who completed the study, six subjects in the NF group and three in the control group were excluded due to excessive artifacts in the EEG. Thus, the final sample consisted of 40 participants in the NF group and 20 in the control group. Demographic and clinical variables for both groups are reported in Table 1. There were no significant differences between groups in any variable.

**Table 1:** **Demographic and clinical variables of the participants at study entry**.

	**NF group *n* = 40**	**Control group *n* = 20**	**NF vs. Control *p*-value**
Gender (male/female)	15/25	4/16	0.24
Age, years (mean ± SD)	53.70 ± 10.87	49.50 ± 10.18	0.16
Antidepressant medication (yes/no)	37/3	18/2	1.00
Comorbidity of anxiety (yes/no)	33/7	15/5	0.51
BDI-II score (mean ± SD)	23.70 ± 13.51	22.25 ± 11.74	0.68
PHQ-9 score (mean ± SD)	13.33 ± 6.84	15.65 ± 5.96	0.21

Between-group differences were assessed by Fisher's exact test and independent samples *t*-tests for categorical and continuous variables, respectively.

### 2.2. Experimental design

**Figure 1:**
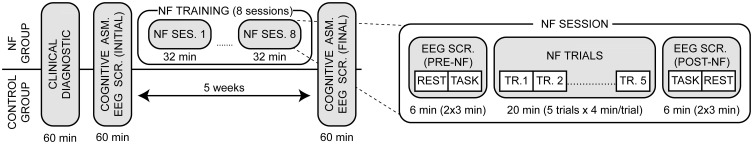
**Experimental design of the study**. After an intake diagnostic both groups executed a pre- and post- cognitive assessment and EEG screening within a 5-week time interval. The NF group performed a total of eight NF sessions (two sessions per week), which were composed of a pre- and post- EEG screening (6 min each) and five training trials (4 min each). The EEG screenings included eyes closed resting state and eyes open task-related activity.

The design of the study is shown in Figure 1. The severity of depressive symptoms was evaluated in a semi-structured interview using the Beck Depression Inventory (BDI-II, Beck et al., 1996) and the Patient Health Questionnaire (PHQ-9, Kroenke et al., 2001). After that, both groups performed a cognitive assessment and an EEG screening at the beginning and at the end of the study (5-week time interval). Cognitive assessments lasted approximately 1 h. The NF group performed eight NF sessions over 4 weeks (two sessions per week). Each session was composed of five trials of 4 min each for a total of 20 min of training, and a pre- and post- EEG screening. For each EEG screening we recorded 3-min of eyes closed resting state activity and 3-min of eyes open task-related activity. In the latter, participants faced a computer screen showing a square that changed saturation color randomly from gray to red or blue (gradually). Participants were instructed to count the number of saturation changes from gray to red as a cognitive challenge (Zoefel et al., 2011). A power EEG analysis and an alpha asymmetry analysis were conducted at the sensor level. Frequency domain standardized low resolution tomography (sLORETA) was used to assess the effect of training at the brain source level. Finally, a correlation analysis between the clinical/cognitive and EEG measurements was conducted at both the sensor and brain source level.

### 2.3. Cognitive performance

Pre- and post- cognitive assessments were carried out at the beginning and at the end of the study. The main outcome variable was WM, which was measured using the Paced Auditory Serial Addition Task (PASAT, Gronwall, 1977) along with the processing speed. This test is sensitive to minimal changes in neurocognitive performance and presents high levels of internal consistency and test-retest reliability (Tombaugh, 2006). The test scores were the number of errors and elapsed time. Episodic memory, attention, and executive functions were also assessed using the following tests: (i) Rey Auditory Verbal Learning Test (RAVLT, Rey, 1964) evaluated episodic memory and the test score was the number of recognized words. (ii) Stroop Color-Word Test (STROOP, Stroop, 1992) evaluated attention and concentration. The test score was the interference, which was standardized across age groups. (iii) Trail Making Test (TMT, Reitan, 1958) evaluated executive functions and was composed of parts A and B. The scores were the elapsed time to complete each part of the test. (iv) Fluency Verbal Test (FAS, Benton and Hamsher, 1976) evaluated verbal phonetic fluency. The test score was the number of evoked words. To determine statistical significance, Two-Way repeated-measures ANOVA (rm-ANOVA) was separately conducted for each score with the between-subject factor Group (NF, Control) and the within-subject factor Time (Pre, Post). Paired samples *t*-tests were performed for within-group (pre vs. post) comparisons.

### 2.4. EEG recording and neurofeedback procedure

EEG data was recorded from 16 electrodes placed at FP1, FP2, F3, Fz, F4, C3, Cz, C4, P7, P3, Pz, P4, P8, O1, Oz, and O2 (subset of the 10/10 system), with the ground and reference electrodes on FPz and on the left earlobe, respectively. EEG was amplified and digitized using a g.tec amplifier (Guger Technologies, Graz, Austria) at a sampling rate of 256 Hz, power-line notch-filtered at 50 Hz and (0.5–60) Hz band-pass filtered. EEG recording and the NF procedure were developed using software of *Bit & Brain Technologies, SL*.

The NF training focused on the increase of the individual upper alpha (UA) power averaged over parieto-occipital locations (P3, Pz, P4, O1, and O2, referred to as feedback electrodes). EEG power was calculated through a short-term fast Fourier transform (FFT) with 1 s hamming window, 30 ms of overlapping, and zero-padded to 1024 points (0.25 Hz resolution). For each session, the pre-NF EEG screening was recorded and then used to calibrate the training for each participant and session. In this calibration step, we automatically filtered out the blinking component from the task-related activity by Independent Component Analysis (ICA), using the FastICA algorithm (Hyvarinen, 1999). Furthermore, we removed the epochs with amplitude larger than 200 μV at any electrode. The Individual Alpha Frequency (IAF) was computed for each electrode on the power spectra of the reconstructed EEG data as the frequency bin with the maximum power value in the extended (7–13 Hz) alpha range (Klimesch, 1999). Note that when no clear alpha peak was found, the IAF was computed on resting state instead. The UA band was thus defined as the (IAF, IAF+2) Hz interval (Klimesch, 1999). Finally, the baseline was computed in task-related activity as the mean UA power averaged across the feedback electrodes, and (5*th*–95*th*) percentiles established the lower and upper limits, respectively. After the calibration, the participants performed the training trials. During online training, EEG data was online filtered from blinking artifacts (through the aforementioned ICA filter) and a visual feedback was then displayed every 30 ms on a computer screen in the form of a square with changing saturation colors. A linear mapping was used to convert between the UA power and the square color. Power values above the baseline were displayed in a red color scale with increasing saturation. Similarly, power values below the baseline were displayed in a blue color scale. The color scales ranged from 0% saturation (baseline in gray color) to 100% saturation in both blue and red color scales set by the lower and upper limits, respectively. No additional information was displayed during training.

Participants in the NF group were instructed to turn the square into red color with the maximum possible saturation, and to maintain it as long as possible. They were not given any mental strategy nor they were aware of the EEG trained parameter. Instead, they were encouraged to try different mental strategies guided by the feedback. To the best of our knowledge, the effect of different mental strategies in the ability to self-regulate brain activity is unclear to date, which is the focus of recent NF studies (Kober et al., 2013).

### 2.5. Offline EEG pre-processing

EEG data from the initial and final EEG screenings was carefully inspected for the presence of artifacts such as eye blinks, eye movements, body movements, and electrocardiogram artifacts. Initially, the extended infomax ICA (Lee et al., 1999) was applied to the task-related activity to remove the eye blinking component. Then, both resting state and task-related activity were imported into EureKa! software (Congedo, 2002) to reject the contaminated data by visual inspection. Participants with at least 30 s of artifact-free data were included in the analysis. EEG spectrum was computed following the same procedure as in the NF procedure. The EEG data of each NF session was cleaned from artifacts using a three-step automatic procedure: filtering of the blinking component by the extended infomax ICA (Lee et al., 1999), epoch rejection by a time-domain threshold (>200 μV) at any electrode, and epoch rejection by a frequency-domain threshold. In the latter step, we computed the power values for each epoch in the bands (1–3 Hz) and (20–30 Hz), commonly affected by ocular and muscular artifacts (Delorme et al., 2007). Then, we converted the log-transformed power values to *z*-scores and removed the outliers (>2.5) at any electrode. Note that the automatic procedure was only used to compute the alpha power in parieto-occipital locations, and no significant differences were found between the two procedures in either resting state [paired samples *t*-test: *t*_(59)_ = −0.77, *p* = 0.44] or task-related activity [*t*_(59)_ = 0.26, *p* = 0.79].

### 2.6. Power EEG analysis in the trained parameter

Power EEG analysis was conducted in the trained parameter: power in the individual UA band, averaged across the feedback electrodes (P3, Pz, P4, O1, O2). The pre-post enhancement was measured as the power change between the initial and final EEG screenings in resting state and task-related activity for both groups. The across- and within-session enhancement were also measured (for the NF group). Across-session enhancement was assessed by a linear trend analysis of the power values in the pre-NF screenings of all sessions and the final screening. The within-session enhancement comprised two measurements, computed in the power values averaged across the NF sessions: a power change comparison between the pre- and post- EEG screenings, and a linear trend analysis of the power values in the pre-NF task-related EEG screening and the five training trials. To determine statistical significance of pre- and post- comparisons, log-transformed power values were entered into a Two-Way rm-ANOVA with the factors Group (NF, Control) and Time (Pre, Post). Paired samples *t*-tests were performed for within-group (pre vs. post) comparisons. Trend analysis consisted in the computation of the slope of a fitted regression line for each participant, and a *t*-test to test the hypothesis of a null slope. The type I error was set at α = 0.05.

### 2.7. Power EEG analysis in the sensor x frequency domain

Power analysis was conducted for all sensors and frequencies in the (1–30 Hz) interval, separately applied for the resting state and task-related activity. The NF training effects were measured as the log-transformed power spectra comparison between the initial and final EEG screening: a between-group comparison (NF vs. control group) on the change power values, and a within-group comparison (pre vs. post) for each group. A cluster-based non-parametric randomization method (Nichols and Holmes, 2002; Maris and Oostenveld, 2007) was used, as implemented in the Fieldtrip toolbox (FC Donders Centre for Cognitive Neuroimaging, Nijmegen, The Netherlands; see http://www.ru.nl/fcdonders/fieldtrip). This method first computes the difference between two conditions by performing *t*-tests in the (sensor, frequency)-pairs. Those pairs exceeding a threshold *q* are clustered on the basis of spatial and spectral adjacency, and then cluster-level statistics are calculated as the sum of the *t*-values within every cluster. The threshold was set to (*q* = 0.05) for resting state and to (*q* = 0.01) for task-related activity. Finally, the significance probability at the cluster-level was estimated by a permutation method (Pesarin, 2001). The distribution of the cluster values was constructed under the null hypothesis by 5000 random permutations and then the observed values were tested against the (1 − α)th percentile of the null distribution. This method controls for the type I error rate and corrects for multiple comparisons both across sensors and frequencies. The type I error at cluster-level was set to α = 0.05.

### 2.8. Alpha asymmetry analysis

Initial and final scores of alpha (8–12 Hz) asymmetry were computed in resting state and task-related activity. EEG data was re-referenced to Cz and asymmetry scores were computed as the normalized power difference between homologous right- and left-side locations, (R−L)/(R+L). See Allen et al. (2004a) for a review on methodological considerations. This score indicates the relative activation of the left over right locations. Thus positive scores indicate left-lateralized activation, i.e., more power over right-side locations due to the inverse relation between alpha power and brain activation (Coan and Allen, 2004). Alpha asymmetry scores were computed in five areas of the scalp: prefrontal (FP: FP2-FP1), frontal (F: F4-F3), central (C: C4-C3), parietal (P: (P7+P3)/2-(P8+P4)/2), and occipital (O: O2-O1). Independent samples *t*-tests were conducted to test for between-group differences in initial scores. We applied *t*-tests on the initial scores (for each group and area of the scalp) to test for null asymmetry scores. Two-Way rm-ANOVA with the factors Group (NF, Control) and Time (Pre, Post) was separately conducted for each area of the scalp to test for pre-post study changes. Bonferroni correction was applied to correct for multiple areas so as to keep the FWER at α = 0.05.

### 2.9. EEG analysis at the brain source level

Frequency domain standardized low resolution tomography, sLORETA (Pascual-Marqui, 2002, 2007) was used to estimate the current density of brain sources in resting state and task-related activity. The current density changes were compared between groups (NF vs. control group) as well as within groups (pre vs. post) for each group. EEG data was re-referenced to a common average reference and Fourier cross-spectral matrices were computed for the following frequency bands: delta (1–4 Hz), theta (4.5–7 Hz), alpha (8–12 Hz), beta1 (12–15 Hz), beta2 (15–20 Hz), and beta3 (20–30 Hz). These bands were defined according to the results obtained in the clustering analysis at the sensor level (Section 2.7). After that, sLORETA estimated the current density values in 6239 voxels (5 mm^3^ spatial resolution). sLORETA applies the boundary element method on the MNI-152 (Montreal Neurological Institute, Canada) template of Mazziotta et al. (2001), and the anatomical labeling is based on probabilities returned by the Daemon Atlas (Lancaster et al., 2000). Finally, current density values were log-transformed and statistical significance of each voxel was determined by a non-parametric randomization procedure using the *t*-max statistic to control the familywise type I error rate (FWER, Holmes et al., 1996). Following this procedure, the null distribution was estimated by 5000 random permutations under the null hypothesis of the maximum absolute *t*-value across all voxels. Then the absolute observed *t*-value for each voxel was tested against the (1 − α)th percentile of the null distribution. Bonferroni correction was applied to correct for multiple bands so as to keep the FWER at α = 0.05.

### 2.10. Correlation analysis: EEG vs. behavioral variables

Spearman correlation was employed to test for a correlation in the initial scores between the clinical/cognitive and EEG variables, as well as in the change scores between the cognitive and EEG variables (clinical variables were not measured after NF training). A total of two clinical variables (Section 2.2) and seven cognitive variables (Section 2.3) were tested. The EEG variables were assessed in resting state and task-related activity and can be divided into two groups: power variables at the sensor level, and current density variables at the brain source level. In both cases, the analysis was conducted in the aforementioned frequency bands (delta, theta, alpha, beta1, beta2, beta3). In the case of the sensor level, power values in each band were averaged across five areas: prefrontal (FP: FP1, FP2), frontal (F: F3, Fz, F4), central (C: C3, Cz, C4), parietal (P: P7, P3, Pz, P4, P8), and occipital (O: O1, Oz, O2). In the case of the brain source level, a randomization procedure (similar to the one used in previous Section 2.9) was used to control the FWER (Holmes et al., 1996). Five-thousand random permutations were performed to construct a distribution of the maximum of the absolute *r*-value across all voxels under the null hypothesis. In both cases the Bonferroni correction was applied to keep the FWER at α = 0.05.

## 3. Results

### 3.1. Cognitive performance

Table 2 summarizes the scores in the cognitive assessments. A significant *Group* × *Time* ANOVA interaction appeared in the PASAT test [# errors: *F*_(1, 46)_ = 5.42, *p* = 0.024; time: *F*_(1, 46)_ = 4.97, *p* = 0.031], showing an improvement in WM performance and processing speed for the NF group only [# errors: *t*_(30)_ = −5.21, *p* < 0.001; time: *t*_(30)_ = −4.91, *p* < 0.001]. Cohen's *d* effect size (Cohen, 1988) revealed a medium-large effect for both the number of errors (*d* = 0.703) and elapsed time (*d* = 0.673). Note that 9 participants in the NF group did not complete the PASAT test in the initial assessment due to excessive cognitive effort, four of whom completed the final assessment. Three participants in the control group did not complete the PASAT test in both initial and final assessments. No significant ANOVA interaction appeared in the other variables. Regarding the within-group (pre vs. post) changes, the number of recognized words increased in the RAVLT test for the NF group [*t*_(39)_ = 3.28, *p* < 0.005]. Interference score in the STROOP test was not significantly modified for any group. Both parts of the TMT test improved for the NF group [part A: *t*_(37)_ = −1.95, *p* = 0.059; part B: *t*_(37)_ = −2.85, *p* < 0.01]. The NF group also improved the number of evoked words in the FAS test [*t*_(38)_ = 2.61, *p* < 0.05]. No significant changes were found for the control group.

**Table 2:** **Cognitive performance at the beginning and at the end of the study for each group**.

**Test**	**NF**			**Control**			**Paired samples *t*-test**		**ANOVA**
**scores**	**Pre mean (s.e.m.)**	**Post mean (s.e.m.)**	**Change mean (s.e.m.)**	**Pre mean (s.e.m.)**	**Post mean (s.e.m.)**	**Change mean (s.e.m.)**	**NF *p*-value**	**Control *p*-value**	***G* × *T p*-value**
**PASAT**									
# errors	13.45 (1.09)	10.13 (1.08)	−3.32 (0.64)	11.59 (2.22)	11.18 (2.21)	−0.41 (1.23)	**<0.001**	0.742	**0.024**
time (s)	253.23 (13.25)	213.68 (12.13)	−39.55 (8.05)	249.76 (20.40)	246.06 (23.77)	−3.71 (16.08)	**<0.001**	0.821	**0.031**
**RAVLT**									
# recognized words	12.25 (0.43)	13.15 (0.30)	0.90 (0.27)	11.55 (0.65)	11.90 (0.50)	0.35 (0.65)	**0.002**	0.597	0.363
**STROOP**									
interference	51.64 (1.23)	53.38 (1.00)	1.74 (1.20)	54.60 (1.80)	54.80 (1.87)	0.20 (1.52)	0.154	0.897	0.443
**TMT**									
part A, time (s)	44.66 (3.03)	38.29 (3.80)	−6.37 (3.26)	41.35 (3.02)	37.95 (2.81)	−3.40 (2.43)	0.059	0.178	0.543
part B, time (s)	101.50 (14.57)	84.87 (11.91)	−16.63 (5.83)	84.90 (11.60)	73.40 (13.51)	−11.50 (10.55)	**0.007**	0.289	0.645
**FAS**									
# evoked words	43.74 (1.87)	46.85 (1.90)	3.10 (1.19)	38.70 (2.68)	39.80 (2.74)	1.10 (1.54)	**0.013**	0.482	0.319

Mean ± s.e.m. scores in pre and post cognitive assessments are shown, as well as the change scores (post-pre). The p-values of the paired samples t-tests are shown for each group (pre vs. post changes), as well as the p-values of the Group × Time interaction in the rm-ANOVAs. Significant effects are marked bold (p < 0.05), statistical trends are underlined (p < 0.1).

### 3.2. Power EEG analysis in the trained parameter

**Figure 2:**
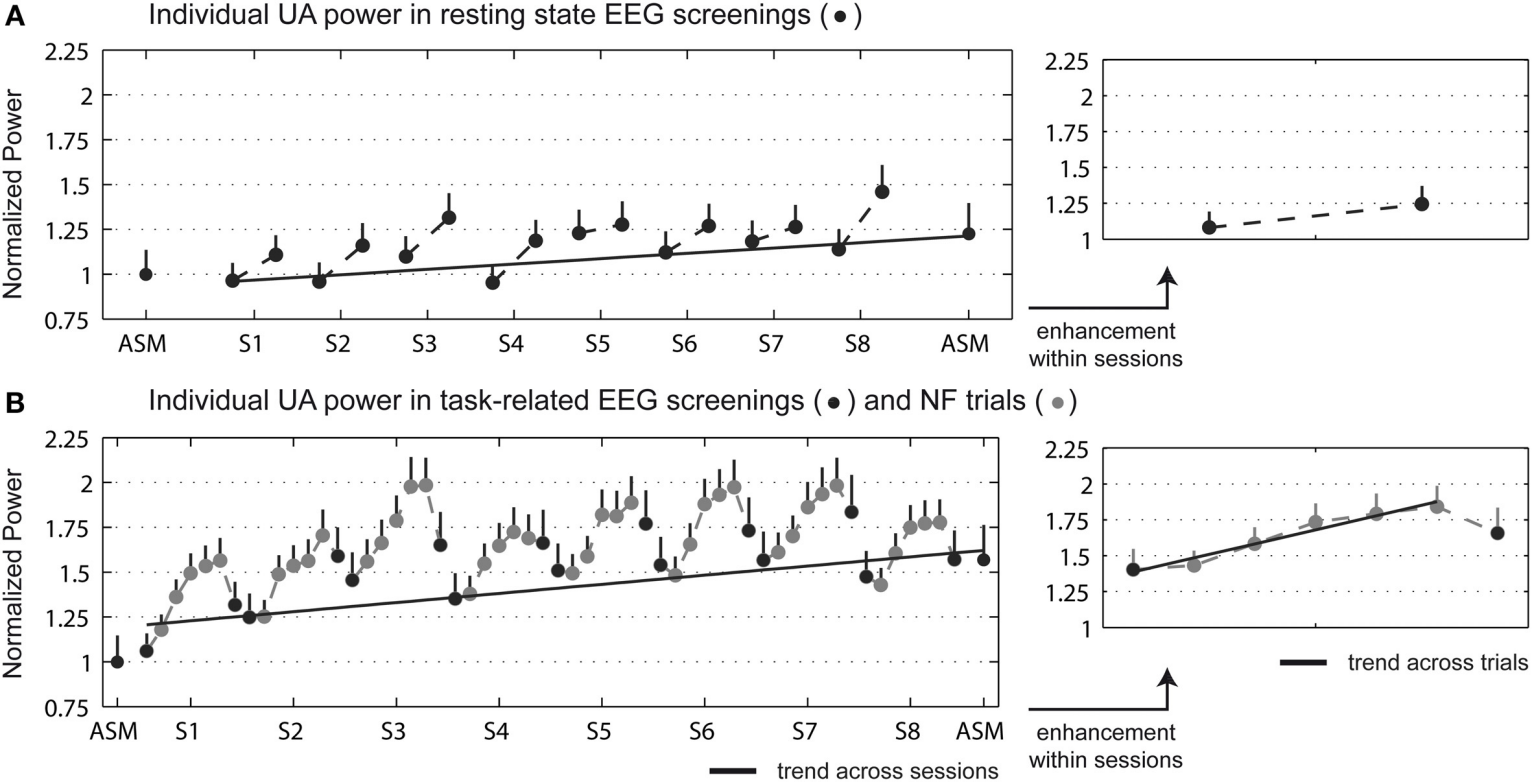
**Individual UA power (mean + s.e.m.) averaged across the feedback electrodes (P3, Pz, P4, O1, O2) for the NF group in the initial and final EEG screenings, and during the NF sessions. (A)** Displays the resting state EEG screenings, and **(B)** the task-related EEG screenings and NF trials. Data was normalized to the power in the initial screening. Right figures display the power enhancement within sessions, in which in the EEG data of the NF sessions was averaged across sessions. Dark and light gray dots depict EEG screenings and NF trials, respectively, and gray line the relevant trend measurements.

The analysis of the pre-post enhancement in the trained parameter (power in the individual UA band averaged across the feedback electrodes: P3, Pz, P4, O1, O2) revealed a no significant *Group* × *Time* ANOVA interaction for the resting state activity. However, a statistical trend between the pre and post measurements was found in the NF group [*t*_(39)_ = −1.72, *p* = 0.093], with an average increase of 22%. Regarding the task-related activity, a significant *Group* × *Time* interaction appeared [*F*_(1, 58)_ = 14.88; *p* < 0.001]. *Post-hoc t*-tests showed a significant pre vs. post difference for the NF group only [*t*_(39)_ = −5.44, *p* < 0.001], with an average increase of 56%. No significant change was found for the control group in either resting state or task-related activity. Note that groups did not differ statistically in initial IAF. Mean ± SD IAF measured in resting state activity was 9.81 ± 0.17 Hz for the NF group and 9.79 ± 0.25 Hz for the control group [*t*-test for independent samples, *t*_(58)_ = 0.075, *p* = 0.94]; in task-related activity it was 9.71 ± 0.19 Hz for the NF group and 9.64 ± 0.24 Hz for the control group [*t*_(58)_ = 0.21, *p* = 0.83]. Furthermore, IAF did not change significantly pre-post the study for either group. The across- and within- session enhancement was measured for the NF group (see Figure 2). Trend analysis revealed a significant UA power increase across the NF sessions in both resting state [*t*_(39)_ = 2.56, *p* = 0.014] and task-related activity [*t*_(39)_ = 4.04, *p* < 0.001]. Regarding the within-session enhancement, a significant power increase between the pre- and post- NF screenings appeared in resting state [*t*_(39)_ = −3.10, *p* < 0.005], with an average increase of 15.1%; as well as in task-related activity [*t*_(39)_ = −5.72, *p* < 0.001], with an average increase of 16.1%. Trend analysis revealed a significant power increase across the NF trials [*t*_(39)_ = 7.81, *p* < 0.001].

### 3.3. Power EEG analysis in the sensor x frequency domain

**Figure 3:**
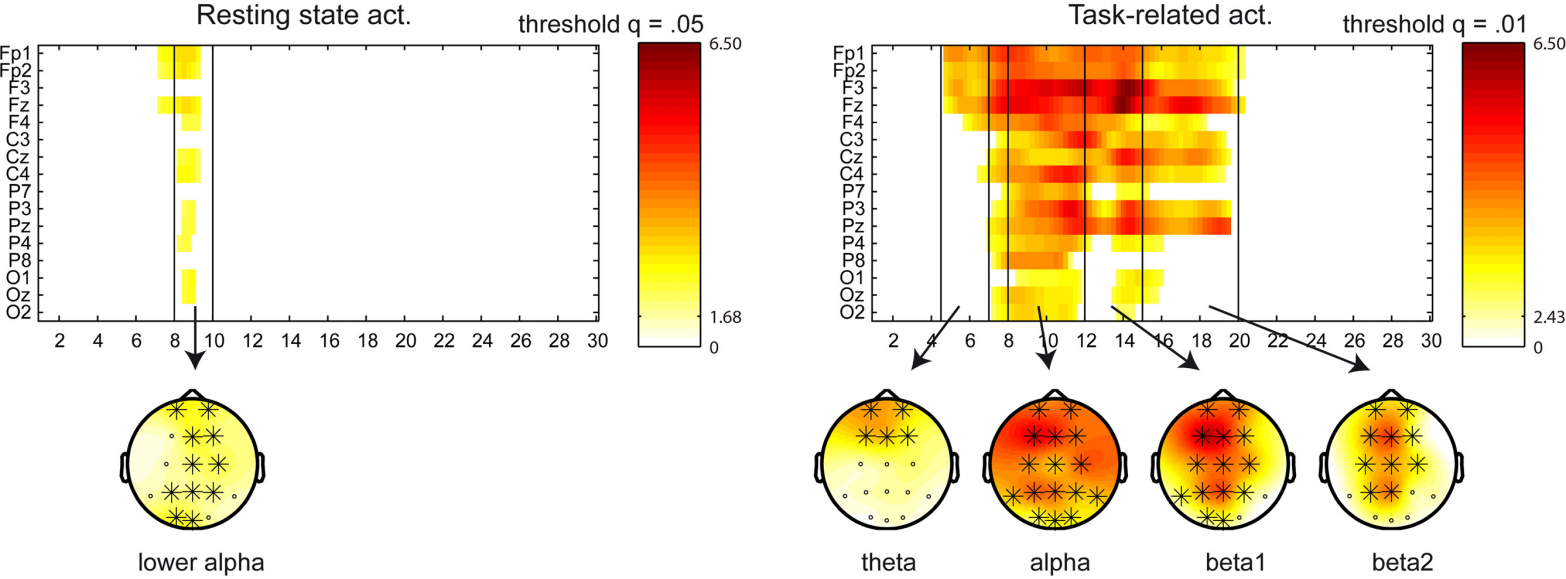
**Sensor x frequency maps displaying the significant clusters (in the power spectra) for the NF group in the within-group (pre vs. post) comparison**. Left figure displays the resting state activity, and right figure the task-related activity. Significant clusters are shown at a given *q* threshold. X axis shows the frequency bins in the (1–30 Hz) frequency range, whereas Y axis shows the sensor locations. Topoplots are displayed in the frequency bands with power changes above the *q* threshold (in significant clusters), and the involved sensors are marked with a cross. Color scale represent *t*-values, indicating a power increase after the NF training.

We have adjusted the frequency bands according to the results obtained in this analysis as follows: delta (1–4 Hz), theta (4.5–7 Hz), alpha (8–12 Hz), beta1 (12–15 Hz), beta2 (15–20 Hz), and beta3 (20–30 Hz). Regarding the power changes in the between-group (NF vs. control) comparison, a cluster appeared in the (5–9 Hz) frequency range for the resting state activity (*p* = 0.008), indicating a power increase for the NF group in theta, apparent in frontal and central locations, and lower part of alpha (8–10 Hz), in frontal, central and parietal locations. A cluster appeared in task-related activity covering the (5.5–12 Hz) range (*p* < 0.0001), indicating a power increase for the NF group in theta, apparent in frontal locations, and alpha, in frontal, central and parietal locations. Figure 3 displays sensor x frequency maps of the power changes (pre vs. post NF training) for the NF group. Note that no significant clusters were found pre vs. post for the control group. Resting state activity showed a cluster in the (7–9.5 Hz) range at a trend level (*p* = 0.073), indicating a power increase in lower alpha in all the scalp areas. A cluster in the (4.5–20 Hz) range appeared for the task-related activity (*p* < 0.0001), indicating a power increase in theta, apparent in frontal locations, alpha and beta1 in all the scalp areas, and beta2, apparent in frontal, central, and parietal locations.

### 3.4. Alpha asymmetry analysis

No significant differences were found between groups in the initial asymmetry scores. We applied *t*-tests on the initial scores (for each area of the scalp) to test for null asymmetry scores within each group. No significant results appeared after strict control of the type I error (see Section 4). Regarding the pre-post changes in asymmetry scores, no significant *Group* × *Time* ANOVA interaction was found in any area of the scalp, as well as no significant pre vs. post differences within each group.

### 3.5. EEG analysis at the brain source level

**Figure 4:**
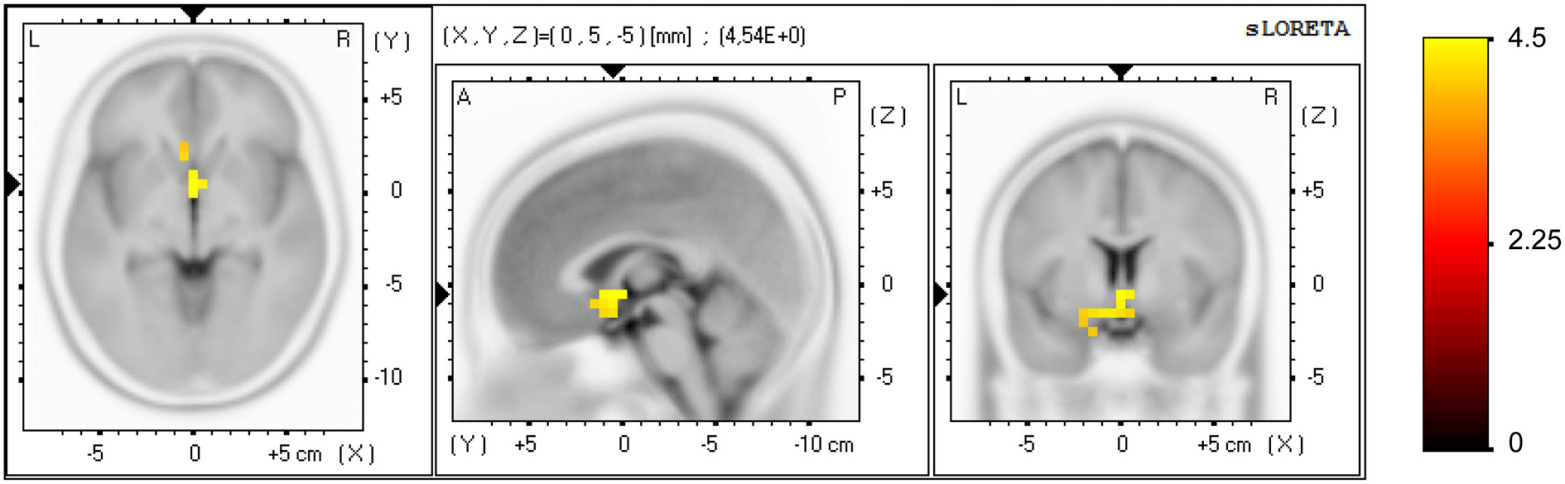
**Effect of the NF training at brain source level for the NF group**. Voxels showing a significant current density increase (pre vs. post NF training) are displayed, measured in alpha band (8–12 Hz) in task-related activity. Axial (left), sagittal (middle), and coronal (right) sections of sLORETA are displayed through the voxel with maximal *t*-value. Color scale represent *t*-values, indicating a current density increase after NF training.

A significant effect was found for the NF group only (pre vs. post NF training) in task-related activity, measured in the alpha band (8–12 Hz), see Figure 4. Forty-two voxels showed a current density increase (threshold *t* = 3.37, α = 0.05). The current density increase was localized in the subgenual anterior cingulate cortex, sgACC (BA 25; XYZ^1^ = 0, 5, −5; *t* = 4.54), subcallosal gyrus (BA 34; XYZ = −10, 5, −15; *t* = 4.35), parahippocampal gyrus (BA 28; XYZ = −15, −5, −15; *t* = 4.13), anterior cingulate cortex, ACC (BA 32; XYZ = −5, 20, −10; *t* = 4.11), and rectal gyrus (BA 11; XYZ = −5, 15, −20; *t* = 4.02).

### 3.6. Correlation analysis: EEG vs. behavioral variables

**Figure 5:**
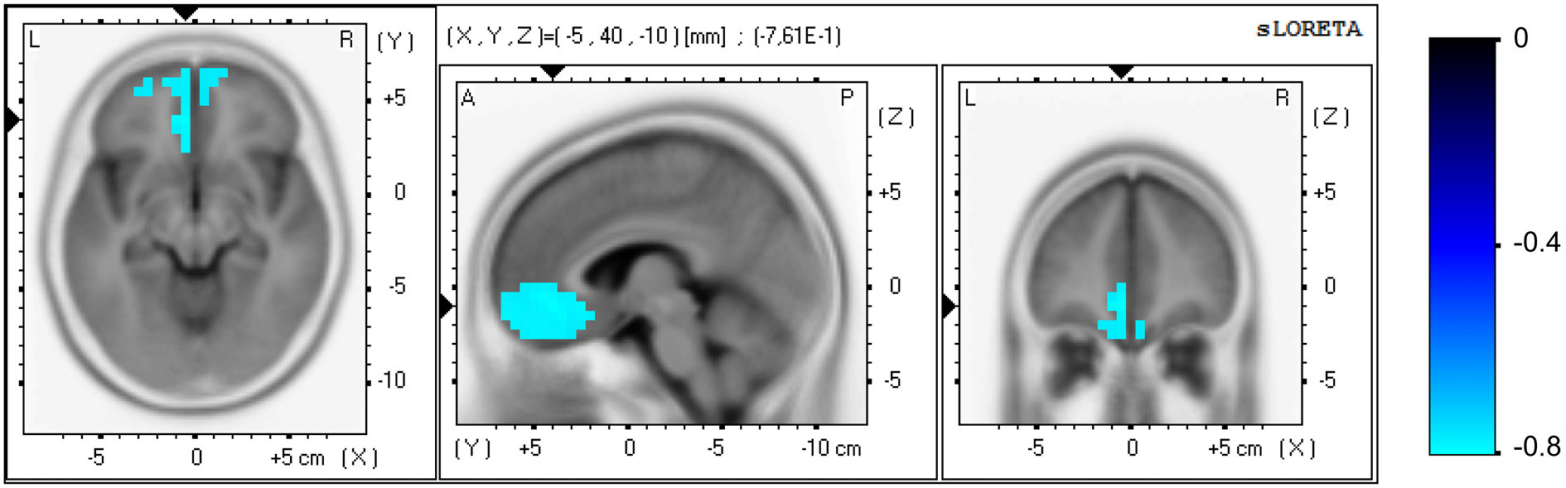
**Correlation analysis for the NF group at the brain source level between the pre vs. post increase in the elapsed time variable of the PASAT test and the increase in current density, measured in beta3 band (20–30 Hz) in task-related activity**. Axial (left), sagittal (middle), and coronal (right) sections of sLORETA are displayed through the voxel with maximal absolute *r*-value. Color scale represent *r*-values, with negative values indicating a positive correlation between the current density increase and the improvement in processing speed (note the inverse relation between the elapsed time in the PASAT test and processing speed).

No significant correlation was found between the clinical and EEG variables at study entry. Significant correlations were found between the elapsed time variable of the PASAT test and EEG variables at both the sensor and brain source level for the NF group only, measured in task-related activity. Regarding the sensor level, a positive correlation appeared in the initial scores between the beta2 (15–20 Hz) power in parietal area (P: P7, P3, Pz, P4, P8) and the elapsed time [*r*_(29)_ = 0.64, *p* = 0.029], i.e., higher beta2 power in parietal locations correlated with slower processing speed. The analysis of the pre vs. post change scores revealed negative correlations between the power increase in each beta sub-band in prefrontal area (FP: FP1, FP2) and the increment in elapsed time: beta1 [12–15 Hz, *r*_(29)_ = −0.68, *p* = 0.007], beta2 [15–20 Hz, *r*_(29)_ = −0.65, *p* = 0.019)], and beta3 [20–30 Hz, *r*_(29)_ = −0.79, *p* < 0.0001]. Thus, the beta enhancement in prefrontal locations correlated with the improvement in processing speed. Regarding the brain source level, the analysis of the pre vs. post change scores revealed a negative correlation between the current density increase measured in the beta3 band (20–30 Hz) and the increment in elapsed time, see Figure 5. One hundred and thirty-three voxels were significant (α = 0.05), which were localized in the ACC (BA 32; XYZ = −5, 40, −10; *r* = −0.761), medial frontal gyrus (BA 11; XYZ = −5, 35, −15; *r* = −0.757), medial frontal gyrus (BA 10; XYZ = −10, 40, −10; *r* = −0.755), pregenual ACC, pgACC (BA 24; XYZ = −5, 30, −5; *r* = −0.747), and subgenual ACC, sgACC (BA 25; XYZ = −5, 25, −20; *r* = −0.741). Thus, the current density increase in the aforementioned regions correlated with the improvement in processing speed. No significant correlations were found for the control group.

## 4. Discussion

The objective of the current work was to explore whether the cognitive symptoms of patients with major depressive disorder (MDD) can be alleviated by EEG-based NF training. Depression is associated with cognitive deficits such as decreased working memory (WM) and attention, among others, which have a clear-cut impact on social and occupational functioning (Austin et al., 2001; Castaneda et al., 2008; Gotlib and Joormann, 2010). We hereby explored the application of a NF protocol based on upper alpha up-regulation to improve WM performance in patients with MDD. The rationale beyond this protocol consists in evidences showing the relation between alpha oscillations and WM performance through inhibitory mechanisms (Klimesch et al., 2007; Freunberger et al., 2011). This NF protocol has obtained promising results in healthy users (Escolano et al., 2011; Nan et al., 2012). Recent evidences suggest that the WM deficits in depression (biases toward negative emotions) may not only be correlates of depression but also increase the vulnerability and recurrence to depression (Gotlib and Joormann, 2010; Levens and Gotlib, 2010). Thus this NF protocol has potential to improve depressive symptoms.

### 4.1. Cognitive performance

WM performance and processing speed (PASAT test) were improved for the NF group in comparison with the control group, showing medium-large effect sizes. While positive findings had been already reported in healthy users (Escolano et al., 2011; Nan et al., 2012) this paper supports the effectiveness of such a protocol in improving WM performance in patients with MDD. Episodic memory, executive functions, and verbal fluency were also improved for the NF group only as revealed by the RAVLT, TMT, and FAS tests. However the improvement in these cognitive functions was not significantly superior to the improvement observed in the control group. These results suggest that the stronger effect of the NF training is specifically found in WM performance and processing speed, whereas the improvement in the latter cognitive functions is marginal and may be explained by an enhancement of cognitive processing as a whole.

### 4.2. EEG analysis

A pre-post enhancement was found in the trained parameter: power in the individual upper alpha band averaged across parieto-occipital locations. The NF group showed an average increase of 56% in task-related activity after the NF training. Resting state activity was also increased for the NF group, with an average increase of 22%, but failed to reach statistical significance in comparison to the control group. No significant changes were found for the control group. In addition to that, upper alpha power in both resting state and task-related activity showed an increase with the number of sessions, as well as an increase (pre vs. post) within each session. Bruder et al. (2008) showed alpha power differences between responders and non-responders to selective serotonin reuptake inhibitor (SSRI), with responders showing greater alpha power in resting state at study entry, specifically measured at occipital locations. While these findings show the potential utility of the present NF protocol in improving the responsiveness to antidepressant medication, it should be confirmed in future studies.

Some NF studies assess the effects of the training not only in the trained parameter, but typically in a small number of pre-determined frequency bands (Escolano et al., 2011; Zoefel et al., 2011; Nan et al., 2012). Here we extended that analysis to assess the power changes in all sensors in the (1–30 Hz) frequency range by a clustering analysis, obtaining sensor x frequency maps of the power changes. We believe that the present analysis can offer a clearer insight of the electrophysiological effects. This analysis was separately applied for the two recording conditions: resting state and task-related activity. Since stronger effects were found in task-related activity in comparison to resting state, we adapted the *q* threshold to each recording condition to get clearer sensor x frequency maps. Please note that *q* threshold does not determine the type I error at cluster-level, which was set to (α = 0.05) for both conditions. Significant clusters were found for the NF group only, showing a power increase after the NF training. The strongest effect in resting state activity appeared in the lower part of alpha (8–10 Hz). Task-related activity showed stronger effects in the (4.5–20 Hz) range, covering theta, alpha, and the lower part of beta. These effects were apparent in anterior, central, and posterior locations. Our results show the common finding that the effects of the NF training on the spectral power are not spatially or spectrally restricted to the trained locations and frequency bands, instead the training engenders profound changes of the homeostatic properties of the brain involving several locations and frequencies (Hughes and John, 1999). The strong effect in task-related activity illustrates the importance of recording EEG in several conditions to provide additional information of the underlying brain processes. This is in contrast to the common practice to study only the resting state, either eyes closed or eyes open.

**Figure 6:**
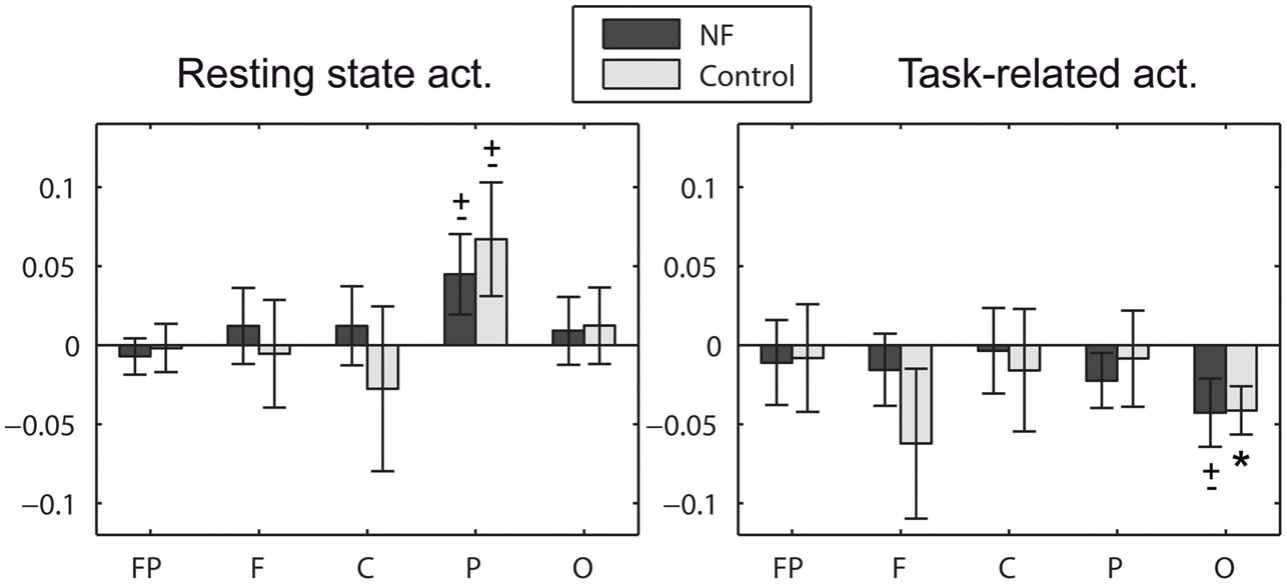
**Initial alpha asymmetry scores measured in prefrontal (FP: FP2−FP1), frontal (F: F4−F3), central (C: C4−C3), parietal (P: (P7+P3)/2−(P8+P4)/2), and occipital (O: O2−O1) areas of the scalp**. Positive asymmetry scores denote left over right activation, i.e., more alpha power in right hemisphere locations. ^*^ significant effect (*p* < 0.05), ± statistical trend (*p* < 0.1). Note that statistical results are not corrected for the multiple areas.

Alpha asymmetry scores did not differ between groups at study entry and it did not change pre-post the study for any group. Note that this study can-not assess the commonly found frontal asymmetry in depression since it involves the comparison with a control group of non-depressive participants (Davidson, 2004). We investigated the initial alpha asymmetry scores for null scores, measured in five areas of the scalp (prefrontal, frontal, central, parietal, and occipital). Strong asymmetry scores were found although they failed to reach statistical significance after strict control of the type I error (Bonferroni correction). Here we summarize the significant results when not correcting for the multiple areas (see Figure 6). Significant scores were found in posterior areas (parietal and occipital). Left-lateralized activation (more alpha power over right locations) was found in resting state in the parietal area at a trend level for both the NF group [*t*_(39)_ = 1.76, *p* = 0.086] and the control group [*t*_(19)_ = 1.86, *p* = 0.077]. These results are in line with previous studies (Kemp et al., 2010). Interestingly, the opposite effect was found in task-related activity. Right-lateralized activation (more alpha power over left locations) was found in task-related activity in the occipital area at a trend level for the NF group [*t*_(39)_ = −1.98, *p* = 0.054], significant for the control group [*t*_(19)_ = −2.71, *p* = 0.014]. Please note that EEG data was recorded using a single earlobe reference, but re-referenced offline to Cz. The most common approach to measure brain asymmetry to date is the use of computer-averaged ears/mastoids reference, or Cz reference (Davidson, 1998; Coan and Allen, 2004; Allen and Cohen, 2010). Although there is no agreement about a preferred montage, reference placement might be a critical issue (Hagemann et al., 2001; Allen et al., 2004b; Davidson, 2004), with unilateral references being discouraged. Thus, the alpha asymmetry effects herein reported should be taken with caution and confirmed in future studies using one of the aforementioned recording approaches.

The analysis at the brain source level (using sLORETA) revealed significant changes in current density after the NF training for the NF group only. The stronger effect was found in task-related activity for the alpha band (8–12 Hz), localized in the subgenual ACC (sgACC, BA 25) extending to the entorhinal cortex (BAs 28, 34), ventromedial prefrontal cortex (BA 32) and orbitofrontal cortex (BA 11). Notice that we are controlling for the familywise type I error rate using a conservative method for correcting also for the multiple frequency bands (*t*-max permutation tests were further corrected using a Bonferroni correction for the multiple bands). Strong effects were also found in other brain structures although they failed to reach statistical significance after such a strict control of the type I error. Here we summarize the significant results when not correcting for the multiple frequency bands, which appeared for the NF group only. Regarding the resting state activity, a current density increase appeared in the pregenual ACC, pgACC (BA 24; XYZ = −5, 35, 10; *t* = 3.25) measured in alpha band. Regarding the task-related activity, a current density increase appeared in the sgACC (BA 25; XYZ = 0, 5, −5; *t* = 3.03) measured in delta (1–4 Hz), in the pgACC (BA 24; XYZ = −5, 30, 0; *t* = 3.01) in theta (4.5–7 Hz), and in the pgACC (BA 24; XYZ = −5, 25, 15; *t* = 3.27) in beta1 (12–15 Hz).

### 4.3. Correlation analysis: EEG vs. behavioral variables

A strong correlation was found between the processing speed (elapsed time variable in the PASAT test) and the power in task-related activity measured in the beta frequency band. The power EEG analysis revealed that higher power in beta2 band (15–20 Hz) in parietal locations positively correlated with slower processing speed at study entry. We also found that the power increase in beta band (specifically in each one of the three beta sub-bands analyzed, ranging from 12 to 30 Hz) in prefrontal locations positively correlated with the improvement in processing speed (pre vs. post NF training). These results suggest that beta band is related to processing speed in different ways according to the area of the scalp involved. Furthermore, a strong correlation was found at the brain source level: the increase in current density measured in beta3 band (20–30 Hz) positively correlated with the improvement in processing speed (pre vs. post NF training). This correlation was localized in the pgACC (BA 24) and sgACC (BA 25) extending to the ventromedial prefrontal cortex (BA 32) and further into the orbitofrontal cortex (BA 11) and frontopolar cortex (BA 10).

Beta band activity has been traditionally linked to somatosensory and motor functions but its functional role is not well understood to date (see Engel and Fries, 2010, for a review). Regarding its perceptual-cognitive role, it has been recently associated with the active continuation of the current cognitive set in tasks involving a strong top-down component (Engel and Fries, 2010). According to this theory, the brain makes predictions in beta oscillations about what it will encounter in the internal and external environment, and updates this by measuring prediction errors, which are encoded by gamma oscillations (Arnal and Giraud, 2012). A large body of research has highlighted the relation of the ACC in error monitoring and prediction of errors (Rushworth and Behrens, 2008; Holroyd et al., 2009). The improvement in processing speed after NF training may be thus explained by this relation.

### 4.4. The involvement of the anterior cingulate cortex

The ACC is known to be linked to cognitive and emotional processes (Bush et al., 2000). Based on cytoarchitecture, lesion, and electrophysiological studies the ACC has been divided into two major functional sub-divisions: a cognitive and an affective sub-divisions (Bush et al., 2000). The cognitive division is localized in the dorsal ACC (areas 24′, 32′), and the affective one is localized in the rostral-ventral ACC (rostral areas 24 and 32, and ventral areas 25 and 33). Functional differences in the ACC between depressive and non-depressive subjects have been repeatedly reported (Mayberg, 1997; Pizzagalli et al., 2001; Davidson et al., 2002). On one hand, decreased activation has been reported in the dorsal ACC (dorsal region of area 32; areas 24′, 32′). On the other hand, increased pre-treatment activation has been found in responders (vs. non-responders) to antidepressant medication in the rostral and ventral ACC (including pregenual areas 24 and 32), and it has been suggested as a predictor of treatment response (Mayberg et al., 1999; Pizzagalli et al., 2001). Pizzagalli et al. (2001) compared depressive participants showing better response to nortriptyline treatment to responders showing worse response. Participants showing a better response had higher pre-treatment theta (6.5–8 Hz) activity localized in the rostral ACC (BA 24, 32), estimated using sLORETA. In this line, the current study showed a current density increase in the rostral ACC (BA 24; XYZ = −5, 30, 0; *t* = 3.01) measured in the theta band (4.5–7 Hz), suggesting that the present NF protocol may increase the response to antidepressant medication. Regarding the correlation between the cognitive and EEG variables, the pregenual ACC (BA 24) and subgenual ACC (BA 25) were positively correlated with the improvement in processing speed. Interestingly, the subgenual ACC has not been traditionally linked to cognitive processing. However, recent studies demonstrate that there is an overlap of autonomic, sensorimotor, affective, and cognitive processing in the midcingulate gyrus (Beissner et al., 2013) suggesting that the functional-anatomical parcellation of the cingulate gyrus is not as simple as has been assumed. The present study suggests that the subgenual ACC, apart from its well-known involvement in autonomic (Beissner et al., 2013) and emotional (Mayberg et al., 1999; Bush et al., 2000) regulation, it also is implicated in cognitive processing. Thus analogous to the midcingulate gyrus also the subgenual ACC may be involved in multiple overlapping functions.

### 4.5. Limitations

Due to the novelty and the exploratory character of the study, the control group designed in the present study was not optimal. On one hand, the number of subjects in the NF and control group was not balanced. We decided to use an allocation ratio 2:1 between the NF and control group. On the other hand, participants were not randomly assigned to the experimental condition nor they were blinded with respect to the experimental condition. Nonetheless, the control group used in the present study can account for practice effects in the cognitive assessments and for electrophysiological changes before and after the study. Regarding the demographic characteristics, around 90% of the participants of the study followed a stable pharmacological antidepressant treatment during the study, 75% of whom consisted either on selective serotonin reuptake inhibitors (SSRI), benzodiazepines, or serotonin-norepinephrine reuptake inhibitors (SNRI). Also, 80% of the participants presented comorbidity with anxiety. Further research is needed to elucidate to what extent the obtained results can be translated to a drug-free population or to a population without anxiety symptoms. The averaged severity of depression was “moderate” for both the NF and control group according to the BDI-II. However, the averaged PHQ-9 scores were slightly lower for the NF group (“moderate”) than for the control group (“moderately severe”). Nonetheless, there were no significant differences between groups in severity of depression as measured by either BDI-II or PHQ-9. The current study estimated the effects at brain source level using sLORETA (MNI-152 template). The spatial resolution and precision of sLORETA method could be improved by computing individual head models based on magnetic resonance imaging (MRI). A comparison of the cognitive performance with healthy subjects would have been an interesting analysis to estimate the significance of the observed effects. This analysis should be taken into account for future studies. Finally, although we have stated that the present NF protocol has the potential to improve depressive symptoms, such hypothesis can-not be corroborated in the present study since clinical variables were not assessed after NF training. Due to the positive cognitive effects, the present NF protocol should be evaluated in future studies using stricter control conditions such as active control conditions (e.g., psychotherapy) or sham feedback. However, a sham feedback control condition may lead to ethical concerns when effective standard treatments are available (La Vaque and Rossiter, 2001).

### Conflict of interest statement

The authors declare that the research was conducted in the absence of any commercial or financial relationships that could be construed as a potential conflict of interest.

## Footnotes

^1^MNI (Montreal Neurological Institute, Canada) coordinates are used throughout this paper.
